# Identification of Suspect-Cerebral Visual Impairment in Children with Special Needs: Moving Towards an Inclusive Diagnostic Process

**DOI:** 10.22599/bioj.462

**Published:** 2025-12-01

**Authors:** Rachel F. Pilling, Sidra Amjad

**Affiliations:** 1 University of Bradford, UK; 2 Bradford Teaching Hospitals NHS Trust, Bradford, UK

**Keywords:** paediatric, children, vision, cerebral visual impairment, examination

## Abstract

**Introduction:**

Cerebral Visual Impairment (CVI) accounts for most visual impairment in children in the developed world. A significant proportion of children in special needs education demonstrate atypical visual function, yet referral thresholds for onward assessments are not defined. We sought to describe the elements of a visual function assessment that might indicate a threshold for referral into CVI diagnostic pathways.

**Method:**

We undertook a retrospective analysis of 50 consecutive records of children attending a specialist visual assessment clinic. Visual ability was documented, including visual acuity, contrast sensitivity, visual field, eye movements, visual attention, accommodation, and optic disc appearance. The aim of this retrospective case note review was to examine the pattern of visual assessment outcomes that raised suspicion of CVI in a cohort of children with special needs.

**Results:**

Atypical response to three or more testing domains was associated with a diagnosis of CVI. Mean age of children was 6.6 years (range 1–16). There was no statistical difference in the mean number of test domains completed by those with/without CVI. (7.3 vs. 8.3; p = 0.5). Children with CVI showed a statistically higher mean number of atypical responses compared with non-CVI patients (4.2 vs. 0.4; p = 0.015).

**Discussion:**

Our findings are in keeping with other studies that indicate that detecting atypical eye movements, visual field, and/or visual attention play a key role in highlighting children who warrant comprehensive assessment for CVI. Facilitating eye health professionals to identify and refer on those children with evidence of CVI-related behaviors will further our endeavors to provide an inclusive approach to diagnosis in a high-risk group.

## Introduction

Cerebral Visual Impairment (CVI) is defined as a verifiable visual dysfunction not attributable to deficits in the anterior visual pathways or any co-existing ocular abnormality (Sakki *et al.*, 2017). It is an umbrella term encompassing a complex interaction of visuomotor, visual memory, and visuo-cognitive functions, which allow the brain to interpret what the eye is seeing. Each child with a diagnosis of CVI will have a unique combination of visual strengths and weaknesses, often accompanied by other neurodevelopmental anomalies. There are no agreed diagnostic criteria for CVI, and formal diagnostic procedures and guidelines continue to be under development.

It is well established that a multidisciplinary approach to CVI diagnosis is appropriate, including involvement of neurodevelopmental paediatric, psychology, and ophthalmology and the wider eye care team (Bauer *et al.*, 2022; Boonstra *et al.*, 2022; NHS Scotland, 2015; Ortibus *et al.*, 2019; Pilling *et al.*, 2022). The diagnostic approach in the UK (Royal College of Ophthalmologists, 2023) suggests that, having excluded ocular causes for visual impairment, a child who fulfills all three of the following criteria is highly likely to have CVI:

1. A neurodevelopmental risk factor.
2. Reported or observed visual dysfunction.
3. Documented atypical visual function on examination.

Parents report persistent difficulties in obtaining a CVI diagnosis and a desire for an earlier diagnosis (Goodenough *et al.*, 2021; Jackel, 2019). Early diagnosis is important for several reasons; those children diagnosed under three years of age have been found to have a better long-term visual prognosis, as implementation of training, habilitation, support strategies, and early intervention can support the child in accessing their vision (Huo *et al.*, 1999). Highlighting visual problems to teachers in special schools has been shown to have measurable benefits for engagement and learning (Blackstone *et al.*, 2022).

NHS England began a national programme of Special School Visual Assessments in 2021, with the aim to assess the visual function of over 120,000 children in special needs education. Outcomes from this and associated pilot services show that between one-fifth and three-fifths of children in special school have one or more areas of atypical visual function (Black *et al.*, 2019; Donaldson *et al.*, 2019; Pilling *et al.*, 2016). There are no published processes to guide clinicians when detecting elements of atypical visual functioning during this testing, in particular the threshold for referring in-hospital eye services for an assessment to diagnose cerebral visual impairment.

A barrier to progressing with this endeavour is a lack of consensus about the nature or extent of ophthalmological examination required of eye health professionals assessing complex needs children for surveillance or diagnosis of CVI. Given the heterogenous nature of presenting signs and features and the high incidence of co-existing medical diagnoses, determining which children should be referred for assessment for CVI in the presence of cognitive, motor, and/or speech delay represents a greater challenge than for major causes of ocular visual impairment (for example, refractive error, amblyopia, or cataract), which rely on routinely performed acuity or refractive assessments.

A specialist clinic for children with complex needs has been in place for over ten years in our institution. The strategy for assessing CVI-related visual dysfunction has evolved to facilitate the engagement of children with a wide range of abilities. A flexible approach, utilising in the main reflexive tests that do not require instruction, speech, or cognitive or motor abilities, enables the detection of visual difficulties no matter the level of the child’s engagement or function (Philip, 2016). Test findings pointing to suspect-CVI are used in conjunction with the community pediatric team assessment to make a formal diagnosis.

This retrospective case note review was undertaken as part of a service improvement evaluation. The aim was to examine the pattern of visual assessment outcomes that raised suspicion of CVI by a consultant pediatric ophthalmologist in a cohort of children with special needs. This in turn would allow more efficient triaging of children and maximize the impact of an oversubscribed clinical resource.

## Materials and Methods

We undertook a review of 50 consecutive clinical records of children attending the Special Needs Vision Clinic between January and July 2022. Data were extracted from the clinical record in December 2022 and were anonymized at the time of collection. Children were referred into this clinic if they attended a specialist nursery or school and/or had an education, health, and care plan (EHCP) recognizing their complex needs and developmental delay. Referrals are received from pediatricians, the visual impairment team in special schools, orthoptists, and ophthalmologists. Patients who have a co-existing structural ocular abnormality (other than optic disc anomalies related to CVI) were excluded from the review.

The special needs vision assessment clinic operates with the ophthalmology department of an NHS teaching hospital trust. All tests were undertaken within the eye clinic environment by a pediatric ophthalmologist (RP). Data relating to the most recent clinical assessment were included. A summary of tests used is shown in Table 1. To maximize engagement, there was no fixed testing protocol; rather, tests were completed in an order determined on a case-by-case basis, led by the individual’s engagement and attention. The specific testing method was selected based on the child’s cognitive ability and attention, as determined by discussion with the parent/carer and the practitioner’s observation of the child’s ability to engage adequately with each test. All tests were performed with both eyes open, wearing a refractive correction if available. A determination of the presence or absence of CVI was made based on the overall impression of the child’s function, as demonstrated using the testing strategy described. A diagnosis of CVI was made when, having excluded co-existing ocular causes, a child with a documented neurodevelopmental risk factor demonstrated visual dysfunction likely to impact on their ability to learn, mobilize, and/or communicate.

**Table 1:** Visual assessment strategy and categorization.


VISUAL FUNCTION DOMAIN	TOOL	NORMAL VALUES/OUTCOMES

Visual Acuity	Bradford Visual Function Box (Pilling *et al.*,2016)	5 mm target or smaller – normal 8 mm target or larger – impaired

Visual Field	Confrontation, using target appropriate for child’s acuity threshold	Visual inattention in one or more hemifields, horizontal or vertical, or global suppression of peripheral field – impaired

Contrast Sensitivity	Hiding Heidi (Elgohary *et al.*, 2017)	2.5% or better – normal 5% or worse – impaired

Eye movements	Tracking at 30–50 cm (using a target appropriate for child’s acuity) Fast relocation movements between two objects at 30–50 cm, help approx. 30 cm apart	Smooth tracking – normal Delayed response, jerky, or hesitant movement – impaired Accurate movement – normal Inaccurate or inefficient movement – impaired

Divided Attention	Using target appropriate for child’s acuity threshold, ability to move attention from an initial target to a newly introduced target	Attention shift within three seconds – normal No shift of attention within three seconds – impaired

Motion Perception	Using target appropriate for child’s acuity threshold, ability to show attention to an object moving horizontally from the midline	Attention shown by eye or head movement, by upper limb reach/grab response, or by stilling of sensory self-stimulation – normal No response shown – impaired

Accommodation	Modified Bell Dynamic retinoscopy using an appropriate sized target at 30–40 cm (Tarczy-Hornoch, 2009)	Brisk change in reflex – normal Delayed or absent change in reflex – impaired

Visual Range	Engagement with own reflection in a mirror moved progressively further away from child	3 m or over – normal <3 metres -impaired (based on age-matched norms and extrapolation to visual acuity) (Bowman *et al.*,2009)

Visual Attention	Using appropriate sized object for acuity, Modified Reaction Time to Fixation (RTF) (Kooiker *et al.*, 2020)	0–1 seconds – normal 2–5 second -impaired >5 seconds – absent

Optic disc assessment	Dilated fundal examination using indirect ophthalmoscope	Typical optic disc appearance – normal Atypical disc appearance (eg disc pallor or PVL-type cupping) – impaired


Data were extracted from the electronic patient record by a research orthoptist (SA) and input into a secure collection tool. Categorical variables were analyzed using chi-squared. Ethical approval was not required for this study.

## Results

Data were extracted from 50 sets of clinical notes. Table 2 presents a summary of patient characteristics and testing outcomes. The mean age of children included was 6.6 years (range 1–16); 58% of participants were male. Thirty-five (70%) children were diagnosed with CVI. A CVI diagnosis was associated with atypical findings in three or more domains. Children found to have CVI were younger than those with non-CVI (5.9 years vs 8.1 years).

**Table 2:** Summary of patient demographics and test outcomes.


	OVERALL	CVI	NON-CVI

Mean Age in years (range)	6.6 (1–16)	5.9 (2–14)	8.1 (1–16)

% Male	58	57	60

Mean number of completed test domains (range)	7.6 (4–10)	7.3 (4–10)	8.3 (4–10)

Mean number of atypical domain responses (range)	3.0 (0–7)	4.2 (1–7)	0.4 (0–2)


**Figure 1:**
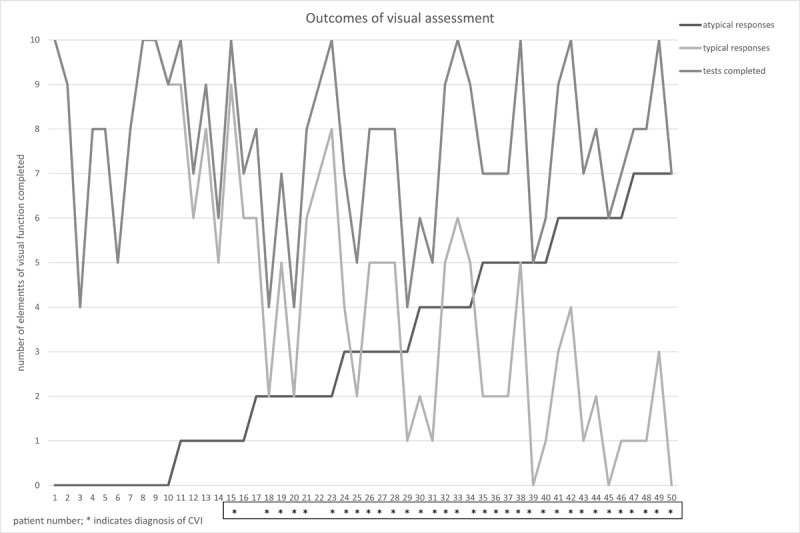
Summary of testing outcomes showing tests completed and number of typical/atypical responses.

Figure 1 summarizes the number of tests completed, the presence of typical/atypical responses, and the diagnosis of CVI. The mean number of domains in which a result was recorded was 7.6 (range 4–10). Children with CVI completed on average fewer test domains than those with non-CVI (7.3 vs. 8.3, p = 0.5). Children with CVI showed a statistically higher mean number of atypical responses compared with non-CVI patients (4.2 vs. 0.4, p = 0.015).

Ten children (20%) had an outcome recorded in all ten visual function domains; of these, five were diagnosed with CVI with an average of 4.8 atypical responses (range 2–7).

Table 3 shows the outcomes for each domain of the visual function assessed. There was a significantly higher proportion of children with atypical responses for all domains in the CVI compared with the non-CVI group (p<0.01 for all domains). Almost all patients had a visual acuity recorded. For both CVI and non-CVI subgroups, over two-thirds of patients could engage for testing of contrast sensitivity, visual field, eye movements, motion perception, divided attention, range, and visual attention. The domains where a response was most difficult to establish were accommodation (60% CVI, 67% non-CVI) and disc appearance (57% CVI, 73% non-CVI).

**Table 3:** Summary of test domain outcomes.


DOMAIN OF VISUAL FUNCTION	TEST COMPLETED % (n)		ATYPICAL RESPONSE % (n)	
	
	CVI	NON-CVI	CVI	NON-CVI

Visual Acuity	91 (31)	93 (14)	51 (16)	14 (2)

Contrast Sensitivity	69 (24)	73 (11)	38 (9)	9 (1)

Visual Field	74 (28)	87 (13)	42 (12)	0 (0)

Eye Movements	82 (29)	100 (15)	86 (25)	7 (1)

Motion perception	69 (24)	93 (14)	46 (11)	0 (0)

Divided Attention	69 (24)	93 (14)	79 (19)	0 (0)

Range	71 (25)	87 (13)	72 (18)	15 (2)

Visual Attention	89 (31)	93 (14)	71 (22)	7 (1)

Accommodation	60 (21)	67 (10)	28 (6)	10 (1)

Disc appearance	57 (20)	73 (11)	20 (4)	9 (1)


Seven children were diagnosed with CVI despite having atypical function in two or fewer domains. Furthermore, six of the children determined not to have CVI were found to have atypical responses in one (four children) or two (two children) domains. Table 4 summarizes these responses. Five of the children diagnosed with CVI (18, 23, 24, 41, and 49), who demonstrated two or fewer atypical features, showed a combination of difficulties in domains related to eye movement, moving objects, and divided attention. These functions of vision are considered to have a significant impact on a child’s ability to access education, learn to read, and mobilize independently. For this reason, children with this pattern of visual dysfunction were offered a CVI diagnosis.

**Table 4:** Contribution of specific areas of visual dysfunction to the diagnosis of CVI with two or fewer atypical features: n = normal response, X = atypical response, – = test not completed/child unable to engage with test.


CHILD NO.	NO. TESTS COMPLETED	CVI DIAGNOSIS	VA	CS	VF	EM	MO	DA	R	ATT	ACC	OD

14	10	no	n	n	n	X	n	n	n	n	n	X

16	7	no	n	–	–	n	n	n	n	X	–	n

18	8	yes	n	n	n	X	–	X	n	–	n	n

23	4	yes	n	n	–	X	X	n	–	–	–	–

24	7	yes	n	n	X	–	n	X	n	n	–	–

26	6	yes	n	n	n	–	–	n	–	X	n	–

29	4	yes	n	X	–	–	–	n	–	–	–	X

25	9	no	n	n	n	n	n	n	n	n	X	–

30	10	no	n	n	n	n	n	n	n	X	n	n

40	8	no	X	X	n	n	n	–	n	n	n	–

41	9	yes	n	n	n	X	n	X	n	n	n	–

46	7	no	X	–	–	n	n	n	n	n	–	n

49	10	yes	n	n	n	X	n	n	n	X	n	n


VA = visual acuity; CS = contrast sensitivity; VF = visual field; EM = eye movements; Att = visual attention; MO = moving objects; DA = divided attention; R = Range; Acc = accommodation; OD = optic disc assessment.

Child 26 had a single atypical feature in the attention domain. This child had fleeting visual attention, which was considered likely to impact their ability to access learning; thus, in the presence of normal acuity, a diagnosis of CVI was made.

Child 29 was able to complete only four testing domains. They were found to have a disc appearance pathognomonic of periventricular leukomalacia and, for this reason, were offered a diagnosis of CVI.

It is apparent that documentation of a single element of visual dysfunction is insufficient to support a diagnosis of CVI. Both Child 16 and Child 30 returned a single atypical result in the attention domain. In both cases, a comment of ‘visual avoidance’ was recorded in the notes, indicating a behavior relating to sensory regulation whereby the child uses vision ‘on their terms’. However, these children were deemed able to self-regulate and adapt to use their vision effectively for the majority of the assessment.

Child 14 returned atypical results in terms of eye movements and disc appearance. The disc appearance documented was disc pallor, indicating a high likelihood of ocular visual impairment. Similarly, Child 40 and Child 46 returned atypical contrast and/or acuity responses, with other domains being normal. Such results would point to ocular impairment. The combination of atypical acuity and contrast is well documented in ocular visual impairment and, in the absence of any visual attentional abnormalities, would not point to CVI. Atypical eye movements or anomalous optic disc appearance alone, in the absence of other areas of visual dysfunction, did not trigger concern about CVI.

The highest number of atypical responses was found in eye movements, divided attention, range, and visual attention. An atypical finding in any one of the domains relating to visual field, motion perception, or divided attention appeared to be a predictor of proceeding to a diagnosis of CVI.

## Discussion

Leaders in the field have stated ‘that tackling CVI is now the biggest challenge and biggest opportunity for reducing the burden of childhood blindness’, and there is a ‘pressing need to look for and document’ visual dysfunction in at-risk groups (Teoh *et al.*, 2021). Assessment of children with special needs to demonstrate areas of visual dysfunction requires a flexible and curious approach. The approach described in this paper would suggest that for a child with documented developmental delay, where a dysfunction is found in three or more areas of visual function (excluding visual acuity and accommodation), a child could be considered suspect-CVI and warrants further assessment.

Recent studies have reported almost 90% of children with profound and multiple disabilities have evidence of atypical visual function (Steendam *et al.*, 2024). Using the testing approach described will support the imminent roll-out of special school eyecare services by offering referral thresholds for in-school optometrists. It may also facilitate expansion of clinic throughput by enabling orthoptists or other eye health professionals to undertake an initial assessment and filter through only those patients reaching the threshold into a multidisciplinary CVI diagnostic clinic.

It is noteworthy that only one in five children completed all testing domains. Subgroup analysis revealed that, while not reaching significance, those with CVI engaged with fewer tests than those without CVI. This conclusion is perhaps expected; children with normal visual function are more able to engage with a fuller range of testing than those with visual dysfunction.

Our study has several strengths. The tests chosen were ‘reflexive’ tests; that is, the child does not require any instruction or direction to perform the test, and it is therefore suitable for children with a broad range of acuities and cognitive abilities. The visual domains and testing strategy offer an approach that has been demonstrated to be implementable not only in a hospital but also in a community setting (Musleh *et al.*, 2024). The testing equipment is inexpensive, widely available, portable, and practicable to use in a range of environments and offers an inclusive approach to assessment.

There are several limitations in the approach we have chosen. Whilst maximizing consistency in reporting, using a single clinician to assess all children may lead to bias in the subjective elements of the testing strategy. It is possible that we have misdiagnosed a child as having normal visual function in the absence of atypical findings. The intention of the clinic is to identify CVI where present, rather than rule out CVI if findings are normal. It is acknowledged that assessment in an eye clinic offers a snapshot of a child’s visual function in a specific environment and may not represent their overall level of function. However, children without CVI will have normal functions in all domains, whatever their environment. The presence of an atypical response should alert the clinician to suspect CVI and prompt further assessment and discussion.

Age-matched norms are not published for all tests; however, chronological age does not confer cognitive age. The youngest child diagnosed with CVI in this cohort was two years old, and by this age, it would be anticipated that in a child with normal visual function development, all domains of visual function in our testing matrix would return a ‘typical’ result. A review of testing protocols used in research to diagnose CVI reports that aside from acuity, the three most commonly included domains were ocular movement (70%), visual field (56%), and visual attentional responses (e.g., blink to light, interaction with objects, and visual fixation) (43%) (McConnell *et al.*, 2020). Our findings are in keeping with this and indicate the same visual domains being key indicators of the presence of CVI.

The clinical assessment protocol outlined does not include a formal history, questionnaires, inventories, or exploration of parent/carer concerns around visual function. These form an essential part of the diagnostic process and can direct the clinician towards the elements of a visual assessment that may be most fruitful in verifying the presence of visual dysfunction.

It is becoming evident that for children with complex needs, CVI need not remain a diagnosis of exclusion, and there is a pressing need to provide a more inclusive diagnostic protocol. Facilitating eye health professionals to identify and refer on those children with evidence of CVI-related behaviors will further our endeavours to provide an inclusive approach to diagnosis in a high-risk group.
